# Detecting Unusual Repetitive Patterns of Behavior Indicative of a Loop-Based Attack in IoT

**DOI:** 10.3390/s24237534

**Published:** 2024-11-26

**Authors:** Asmaa Munshi

**Affiliations:** College of Computer Science and Engineering, University of Jeddah, Jeddah 21959, Saudi Arabia; ammunshi@uj.edu.sa

**Keywords:** malicious attacks, detection of attacks IoT loop-based attacks

## Abstract

Given the high risk of Internet of Things (IoT) device compromise, it is crucial to discuss the attack detection aspect. However, due to the physical limitations of IoT, such as battery life and sensing and processing power, the widely used detection techniques, such as signature-based or anomaly-based detection, are quite ineffective. This research extracted loop-based cases from the transmission session dataset of “CTU-IoT-Malware-Capture-7-1” (“Linux, Mirai”) and implemented a loop-based detection machine learning approach. The research employed nine machine learning models to illustrate how the loop patterns of the datasets can facilitate detection. The results of this study indicate that the XGBoost model achieves the best performance in terms of “Accuracy: 8.85%”, “Precision: 96.57% (Class)”, “Recall: 96.72% (Class 1)”, and “F1-Score: 6.24%”. The XGBoost model demonstrated exceptional performance across all metrics, indicating its capability in handling large IoT datasets effectively. It provides not only high accuracy but also strong generalization, which is crucial for detecting intricate and diverse patterns of malicious behavior in IoT networks. Its precision and recall performance further highlight its robustness in identifying both attack and normal activity, reducing the chances of false positives and negatives, making it a superior choice for real-time IoT threat detection.

## 1. Introduction

Loop-based malicious attacks in IoT exploit vulnerabilities in devices or networks via repetitive, cyclical actions [1]. These activities disrupt operations, exhaust resources, or inflict further damage [2]. Researchers have established that loop-based malicious attacks execute repetitive malicious requests or commands, not only to overwhelm the system but also to exploit vulnerabilities [3]. Numerous adoptions of IoT in human activities have demonstrated loop-based attacks as the most effective way to exploit vulnerabilities [4,5,6]. The penetration of IoT devices is increasing swiftly, and the security issues associated with these devices are generating significant concerns [7]. It’s crucial to acknowledge that creating IoT devices with security issues is a common obstacle [8]. However, looping in IoT devices presents a technical issue that is linked to the system’s programming codes [9]. Almost all loop-based cyberattacks stem from the functionality of the codes that govern the IoT devices [10]. The failure of IoT devices in this case will result in downtime for end users of the IoT network [11]. Lastly, the failure of IoT devices can generate significant traffic, potentially overwhelming network infrastructure [12].

The research problems highlighted by this study stem from the fact that only a limited number of studies have examined the operational behavior of loop-based malicious attacks [13,14,15,16]. We also observed that loop-based malicious attacks in IoT can manifest as “Repetitive Actions”, “Resource Exhaustion”, “Disruption of Normal Functionality”, “Exploitation of Communication Protocols”, and “Triggering Software or Hardware Vulnerabilities” [17]. While an IoT device or system that receives persistent malicious commands or requests from attackers may result in system degradation and other adverse effects [18,19], persistent looping attacks can impair the performance of IoT devices [20]. A specific instance of these adverse effects is that an attacker may continuously toggle lights, lock and unlock doors, or alter temperature settings in a smart home, resulting in disorientation and irritation [21,22]. Similarly, repeated buffer overflow in an IoT network transmission session might lead to device malfunction or compromise [23].

Considering the issues raised that are associated with “Loop-based Malicious Attacks in IoT”, this current research explores those concerns, specifically conceptualized loop-based attack detection, and contributes in the following ways:

- The research was able to demonstrate that the XGBoost model has an excellent discriminative ability, effectively distinguishing between normal and malicious loop-based attacks in IoT.
- The research was able to establish that Loop-based attacks are a Threat to IoT Systems based on the fact that “Resource Constraints” of most IoT devices have limited computing, memory, and energy resources. Loop-based attacks can quickly drain these resources, making the devices non-functional [23]. Similarly, a widespread impact can emerge as IoT devices are often interconnected in smart environments (e.g., smart homes, factories), a loop-based attack on one device can potentially affect the entire IoT ecosystem, causing a cascading failure or system-wide disruption. Another issue lies with “Limited Security” as many IoT devices lack advanced security features due to their constrained nature, making them more vulnerable to simple, repetitive attacks [24,25].
- Finally, the research was able to establish that “Real-time Operations” is an IoT systems that is often designed for real-time operation, such as controlling industrial machinery, healthcare devices, or smart city infrastructure. A loop-based attack that disrupts real-time communication can lead to safety risks or service interruptions.

Below is a description of the subsequent sections in this paper: Section 2 presents the related work. Section 3 outlines the models utilized for this study. Section 4 presents the research methodology, followed by the experimentation and results obtained. Section 6 presents the discussion and future work, and finally, Section 7 concludes the research study.

## 2. Related Work

There are many previous research studies associated with “systematic reviews” and general surveys within IoT that provide valuable insights on the current state of IoT applications. Among the crucial studies associated with this is the work of Imran et al. Imran et al. [1] conducted a comprehensive review of the existing literature and presented challenges in IoT and sensor networks. Al-Hadhrami and Hussain [2] undertook a systematic literature review that compiles research on Distributed Denial of Service (DDoS) attacks in IoT networks. Similarly, Tahsien et al. [3] conducted a survey of machine learning techniques used in IoT security applications. Mishra and Pandya [4] conducted a systematic review of IoT applications and security challenges in another research study. Additionally, Mittal et al. [7] conducted a systematic review of deep learning methods designed for detecting DDoS attacks in IoT environments. All this previous work requires significant computational resources, which can be a limitation for IoT systems.

Numerous research studies are also involved in the design and development of IoT security, with a focus on its implementation. Zhou et al.’s work [13], which designs and evaluates an intrusion detection system for heterogeneous IoT networks, is crucial in this regard. The proposed system effectively identifies attacks in complex IoT environments, though its performance varies depending on the heterogeneity and size of the network. Similarly, Arshad et al. [14] proposed a framework and conducted simulations to evaluate their IoT performance framework. The results effectively detect intrusions while minimizing energy consumption, making the framework suitable for IoT devices with limited resources. Another study from Alhowaide et al. [18] presents an ensemble-based model and evaluates its performance through experimental validation. The model demonstrated improved accuracy and reduced false positives in detecting IoT-based intrusions when compared to individual detection methods. The study of Reza et al. [22] used a real-time detection approach and evaluated it through experiments. The findings indicate that the real-time detection system provided timely and accurate intrusion alerts, improving response times in IoT security scenarios. Another study by Noman and Abu-Sharkh [25] combines a comprehensive review of code injection attacks with practical implementations and analysis. The paper demonstrated that code injection attacks pose a major threat to wireless IoT systems, with severe consequences such as denial of service (DoS) and data breaches.

Patel et al. [26] offered a dynamic loop-based methodology to enhance the robustness of wide-area damping control systems against cyberattacks. The authors employ control theory and dynamic feedback mechanisms to develop a system capable of detecting and alleviating cyberattacks that threaten power system stability. The methodology entails modeling the cyber-physical system and integrating redundancy to endure harmful interferences. The proposed solution effectively improves the resilience of wide-area damping controls, demonstrating that the system can sustain stability even amid cyberattacks. The dynamic loop methodology successfully alleviates delays and disturbances resulting from attacks, enhancing the reliability of power grid operations during adverse conditions.

Shang et al. [27] proposed a network design model employing a loop-based methodology for the allocation of defense resources in IoT systems amid uncertain environments. The authors quantify the uncertainty in network operations and include it in the resource allocation model, employing game theory and optimization to develop a resilient system against future cyber threats. The loop-based network design paradigm enhances the efficacy of defense resource allocation in unpredictable IoT environments. The results demonstrate that the approach improves the system’s resilience against unforeseen attacks by carefully distributing resources to critical areas, thereby diminishing the system’s susceptibility.

Oruganti et al. [28] specifically designed a hardware-in-the-loop (HIL) testbed to evaluate the cybersecurity of automotive embedded systems. The testbed facilitates realistic evaluation of security vulnerabilities and the effects of cyberattacks on automotive systems by integrating physical hardware components with virtual settings. The HIL testbed demonstrates efficacy in identifying vulnerabilities within automobile embedded systems. It facilitates a comprehensive evaluation of cybersecurity protocols, offering a regulated setting to replicate assaults and assess system reactions, which is essential for enhancing the security of automotive systems.

For the cyber–physical layer of the smart grid, Gupta et al. [29] presented an intrusion detection system (IDS) that uses an artificial neural network (ANN) based on loop-based learning methodologies. The model perpetually observes network traffic and identifies irregularities suggestive of cyberattacks instantaneously. The loop-based artificial neural network method enhances detection accuracy and diminishes false positives in the identification of cyberattacks on the smart grid. The intelligent loop-based learning approach improves the adaptability of the IDS, increasing its efficacy in identifying various intrusions.

Brindha Devi [30] developed optimized deep learning techniques for IoT attack detection and mitigation. The optimized deep learning models achieved high accuracy in detecting various types of attacks, including DDoS and data breaches, with improved computational efficiency. The study highlights the importance of model optimization for resource-constrained IoT devices.

Alangari [31] introduced an unsupervised machine learning algorithm for detecting attacks and anomalies in IoT sensor data. The unsupervised learning approach was effective in detecting unknown and evolving threats without labeled data. The model showed promise in identifying anomalies in IoT sensors, enhancing detection in scenarios where supervised learning models fall short. Paganraj et al. [32] proposed a machine learning-based technique (DAIR-MLT) for detecting and avoiding routing attacks in IoT networks. The DAIR-MLT model significantly reduced routing attacks, such as blackhole and grayhole attacks, improving overall network performance. The study emphasizes the importance of integrating such models into IoT network protocols to enhance security. Kumar and Singh [33] employed reinforcement learning to develop a DDoS detection and prevention system for edge computing environments in IoT networks: The reinforcement learning model showed significant success in mitigating DDoS attacks, especially in edge computing scenarios where resource constraints are critical. The system dynamically adapted to network changes, reducing false positives and improving attack response times.

The review of research regarding IoT network security underscores the efficacy of machine learning and deep learning models in identifying cyber-attacks, while also exposing significant limitations, including restricted generalizability, dependence on labeled data, absence of real-time application, susceptibility to adversarial attacks, and energy inefficiency in resource-constrained IoT settings. Future research must to concentrate on creating generalizable, unsupervised, and lightweight models that exhibit resilience to adversarial threats and can function in real-time without high energy consumption. The exploration of federated learning and comprehensive threat detection across diverse attack vectors is essential for improving IoT security. Devi et al. [30] exhibited superior performance, as their optimized deep learning model achieved a balance between detection accuracy and computational economy, rendering it advantageous for actual IoT applications.

## 3. The Architectures of the Models

More and more organizations rely on machine learning to protect their systems. Several algorithms have been used in loop-based counter-attack systems in recent years, but selecting the most effective algorithm remains a challenge due to the different types of applications and systems [34]. This research adopted the following machine learning algorithm: “Logistic Regression”, “Random Forest”, “XGBoost”, “Support Vector Machine”, “Neural Network”, “K-Nearest Neighbors”, “Decision Tree”, “Naive Bayes”, and “AdaBoost”. The justification for the selection of the nine models start from the analysis of the problem to the model selection. While loop-based malicious attacks in IoT manifest as repetitive actions, which leads to resource exhaustion, this can be linked to machine learning ability for learning repetitive events. Machine learning can provide a detection scene towards persistent malicious commands or requests from attackers that may result in system degradation and other adverse effects for persistent looping attacks which can impair the performance of IoT devices.

The classification and features of these models are selected based on their good performance in prediction and detection capabilities. Furthermore, classical networking security mitigation techniques can become inefficient due to their manual nature or due to the inability to simultaneously deploy in real-time on a specific network. To remove these limitations, machine learning could become an efficient method of improving network security by actively monitoring patterns and making predictions or taking actions when certain patterns induce harmful behaviors [35]. In the context of computer networks, ML models are used to classify and accelerate arbitrary input data to reduce or prevent the output of critical events or data from crossing predetermined thresholds [36]. Specifically, loop detection refers to the capability of the algorithm to predict the characteristics of the network traffic after being trained with ordinary events and behavior recordings, allowing a network to isolate more immaculate traffic and minimize the time to respond to security vulnerabilities [37,38].

### 3.1. Logistic Regression

These are some of the characteristics of logistic regression: It handles non-linear relationships as it utilizes a transformation function to find the best fitting model to predict the binary outcome over the independent variables. Even if the binary outcome is assumed through a linear model in maximum likelihood, logistic regression results would still be accurate. For the responses that are jointly caused, independence between the residuals need not be. These are some of the characteristics of logistic regression: it handles non-linear relationships as it utilizes a transformation function to find the best fitting model to predict the binary outcome over the independent variables [39]. Even if the binary outcome is assumed through a linear model in maximum likelihood, logistic regression results would still be accurate. The logistic regression model estimates the probability that a given instance belongs to a class y=1. The probability is modeled using the logistic (sigmoid) function presented in Equation (1) [39]:

(1) $$P\left(y=1|x\right)=\frac{1}{1+e^{-\left(\omega x+b\right)}}$$

where ω is the vector of weights, x is the input feature vector, and b is the bias term. The cost function for logistic regression is the binary cross-entropy (or log-loss) presented in Equation (2) [39]:

(2) $$J\left(w,b\right)=-\frac{1}{m}\sum_{i=1}^{m}\left[y_{i}log\left(h\left(x_{i}\right)\right)+\left(1-y_{i}\right)log\left(1-h\left(x_{i}\right)\right)\right]$$

where $h\left(x_{i}\right)$ is the predicted probability of the sample i. Finally, the gradient descent update is resolved by Equation (3) [39]:

(3) $${\;w}_{j}=\sigma \frac{\tau J\left(w,b\right)}{{\tau w}_{j}}$$

where σ is the learning rate and $\frac{\tau J\left(w,b\right)}{{\tau w}_{j}}$ is the gradient of the cost function with respect to $w_{j}$. $\frac{\tau J\left(w,b\right)}{{\tau w}_{j}}$ is the partial derivative of the cost function $J\left(w,b\right)$ with respect to $w_{j}$; this indicates that $w_{j}$ is updated by subtracting $\sigma \frac{\tau J\left(w,b\right)}{{\tau w}_{j}}$. This process is repeated iteratively for each weight $w_{j}$. τ represents the current step in the iterative optimization process. At each step *t* the weight $w_{j}$ is adjusted to gradually minimize the cost function $J\left(w,b\right)$.

### 3.2. Decision Tree

Decision trees are employed in aiding both binary and real classification tasks. They are created by recursively dividing the dataset according to the attribute related to the dataset; the missing value case is solved by inserting testing patterns placed at each leaf node for both branches of a node. The tree stops growing when the leaf does not contain either the patterns left or when it contains entirely a single purpose. However, the stopping method should also be included, otherwise, the overfitting problem might be encountered [40,41]. For that, the pruning concept is usually applied to the tree regarding a concept known as Minimal Description Length, which is essentially an application of this principle to decision trees.

In a tree representation, two outcomes or branches and results in the appropriate function covering the sub-condition. Additionally, an internal node represents a test on a specific attribute, and branches from the node indicate the results of the test. Lastly, leaf nodes represent decision alternatives. Trees are commonly used for many machine learning problems with the greatest benefit [42]. They are devised by partitioning the higher to the lower branch of the tree based on a given attribute at each line. An entropy function in any given dataset *S* is the only classification trees that represent the simplest form of decision trees by Equation (4) [42]:

(4) $$S=-\sum_{i=1}^{n}p_{i}{log}_{2}p_{i}$$

where $p_{i}$ is the probability of class i in dataset S. This proceeds with information gain *A* or the features, where sharing information on how a set of characteristics of the dataset is structured in Equation (5) [42]:

(5) $$S,\;A=S=-\sum_{v\in A}\frac{\left|S_{v}\right|}{\left|S\right|}\left(S_{v}\right)$$

where $S_{v}$ is the subset of S and A is the features value v. These structures are mainly the splitting trees since the nodes are divided into two or more subsets by attributes. In advance, when the amount of predictors is detected as independent of the particular target variable, predictions are made by the most popular level of the terminal in the ideal example in the partitioned pattern recurrence.

### 3.3. Random Forest

Random forest is a popular machine learning algorithm mostly used in classification and function approximation problems. This algorithm is an extension of the classification and regression tree decision forest learners [43]. The algorithm requires a number of parameters to be set during its creation. Although it has been widely used, our initial analysis indicates that a poor choice of parameters can badly impact the learning phase. In this section, we explain the random forest parameter choice and searching algorithm we implemented to achieve good performance in the detection of loop-based DDoS attacks. Random forest constructs a number of decision trees based on bootstrap input data [44]. Each model is trained on a subset of predictor features available for the dataset, called features bagging. Each training process is repeated recursively for a predefined number of levels in the tree. When comparing with the regular decision tree, the difference in random forests is that during a split, each candidate feature is chosen randomly, and the best one is selected among them. This feature criterion is used to narrow the trees down to a small depth.

In the training and bagging of trees process, the algorithm requires different parameters to be defined by the system implementer. One parameter to choose is the number of candidate features randomly selected in each split. In the context of the monitored dataset, not all features are equally important [45]. Thus, it becomes relevant to measure the average importance of all features and focus on the most important ones. While the decision tree selected the available branches, in “Random Forest” the decision to select branches lies with randomly selecting sample subsets of the original dataset with replacements to create multiple bootstrapped datasets [46]. For each bootstrap sample, a decision tree grows an average prediction of all decision trees to get the final prediction, which is calculated by Equation (6):

(6) $$\tilde{y}=\frac{1}{n}\sum_{i=1}^{n}f_{i}\left(x\right)$$

where $\tilde{y}$ is the predicted output, and *n* is the number of decision trees in the “Random Forest”. $\sum_{i=1}^{n}f_{i}\left(x\right)$ is the summation over all *n* in the forest for prediction from ith decision tree in the “Random Forest” for the input *x.*

This research follows the selection procedure suggested by various algorithms that are able to remove irrelevant features, conserving the important information of the dataset. They produce a reliable propagator for the most important features. The research also varied the number of repetitive assignments of features.

### 3.4. Neural Network

There are two types of neural network for classifying sequences or other data in dimensions higher than one. Feedforward neural networks are the first of these. As their name implies, such networks contain no back loops [47]. Learning in these types of network makes use of methods for which learning inputs are first designated for each association between network inputs and desired responses. In the second type of neural network, connections between neurons form either complete loops or sub-loops. The output data sequence or set point is used to determine the supervised target value for a given set of input data [48]. The system is trained using the fixed input-output values, allowing the creation of internal dynamics that result in the desired network output. Recurrent neural networks can estimate sequence relationships which, along with their ability to utilize contextual information in decision and problem-solving stages, make them particularly appropriate for detecting loop execution attacks [49]. A recurrent or back-looped neural network can be considered a system that processes the input error signal produced at its next time step. Errors in processing the input signals result in adjustments of the neural network’s synaptic weights. When recurrent neural networks learn, they optimize both the network’s and critter’s output behavior simultaneously over time. Because recurrent logic typically behaves on a timescale longer than that of feedforward logic, recurrent neural networks can develop highly useful logical structures that provide extensive optimization benefits [50]. For a single neuron, it is represented in Equation (7):

(7) $$z=\sum_{i=1}^{n}w_{i}x_{i}+b$$

where $w_{i}$ are weights, $x_{i}$ are inputs, and b is the bias term. Activation Function: commonly, the sigmoid or ReLU function.

### 3.5. Support Vector Machine (SVM)

SVM is one of the older forms of machine learning, and it is used to solve problems in the supervised learning domain. In essence, SVMs identify the linear separation that is most valid for the two classes of data under study. It then uses this to classify different classes of data. This has also been explored for multi-class categorization [51]. A technique that uses a one-vs-all method has been quite popular and successful in this context.

In the case of SVM, all input data are first mapped to a multidimensional space. Therein, the proper separation by a hyperplane is then sought. For the case of two-dimensional separation, it is commonly referred to as a linear classifier. Another feature of the SVM classifier is that it can adjust well to high-dimensional spaces, e.g., when data are linearly inseparable in lower dimensions. The popularity of SVM lies in its ability to be effectively used in such cases. Lastly, SVM is important when the classes being dealt with are imbalanced [52]. This refers to situations where one class contains significantly more elements than the other class. It should be noted that most learning algorithms are quite successful when data contain equal numbers of data points to represent the different classes.

### 3.6. K-Nearest Neighbors

The K-Nearest Neighbors (KNN) algorithm is a non-parametric learning model that classifies attacked and unattacked instances based on their feature similarity and comes under the supervised category of machine learning [53]. Note that the algorithm uses the data for training applied in scalar form to make predictions. The predictions are made for a category based on an assumption known as ceteris paribus [54]. For example, for K = 1, we check for the nearest neighbor for voting that category. If K > 1, we look for vote counts and pick the category with the highest count. The distance computed is the Euclidean distance. Unfortunately, the KNN algorithm performs faster during training than testing due to the relatively slow process of computing the distance [55].

The K-Nearest Neighbors algorithm can be improved further computationally by using Ball Trees or KD-trees. Such changes in algorithms allow KNN to have a faster run time. KNN, along with other classifiers’ performances, can be tested by using various libraries [56]. Through the iteration of the nearest neighbors to determine the voted category, the algorithm can tell the distance each data point is from the rest as well as itself. These distances associate the data with the category they belong to [57]. This is useful as the generative machine learning model produces data likely to belong to its own category.

### 3.7. Naive Bayes

Naive Bayes (NB) is one of the simplest, fastest, and most often used algorithms for creating models. It is a probabilistic classification algorithm inspired by Bayes’ Theorem. NB is based on the assumption that input variables are independent features, which is why it is called Naive Bayes. Even if input features are correlated or dependent, this algorithm still works well. In this algorithm, an assumption of independence between every pair of features is made [58]. It chooses the class with the highest probability after applying Bayes’ theorem to build a model.

There are two types of Naive Bayes: Multinomial and Gaussian. Gaussian NB uses the normal distribution for continuous attributes because it models the number of occurrences or a count, which is why it is used for classification models regarding qualitative data [59]. Despite normality assumptions, Gaussian NB works well, especially on large problems, because of Naive Bayes’ implementation of parametric learning and the simplicity of its derived decision boundary.

### 3.8. XGBoost

XGBoost is an improved version of an earlier modeling system called gboost. XGBoost greatly improves on gboost. XGBoost is particularly important because it allows for the use of multiple processors during execution. This makes it feasible to train XGBoost models, even for very large machine learning problems in relatively short periods of time. The term XGBoost is short for eXtreme Gradient Boosting [60].

The basic idea behind gboost and XGBoost is gradient boosting. In gradient boosting, we first fit a simple model to our data. We then use the residual, i.e., the actual observed raw data minus the predicted values from the simple model as inputs to then fit another model. We then use the residuals from both of the first two fits as inputs to another model, and we repeat the process, using residuals from the previous model fit as inputs for the next model fit [61]. This procedure keeps going, often through the use of hundreds, even thousands of model fits. In the end, we add the predictions gained by the very simple model to each of the model predictions gained through the process. This gives us our final model output. The system is called gradient boosting because positive residuals get positive weights, while negative residuals get negative weights, with the predictions tending to slowly converge towards the observed values.

### 3.9. AdaBoost

AdaBoost stands for Adaptive Boosting, which is a machine learning meta-algorithm. Essentially, it is a boosting method used to enhance performance by combining multiple individual ‘weak’ learning models to form a ‘strong’ ensemble. AdaBoost can be used with other machine learning algorithms and has been used extensively to improve and facilitate a wide range of attempts at solving real-world problems [62]. In ‘classification’ problems, AdaBoost typically uses decision trees as the original learner. Then, it chooses the type of weak classifier (decision tree) as a primary one.

Adaboost is selected for the loop-based attack detection because of its capability to combine several “weak learners” to create a final prediction. Because of its weight feature and feature selection, Adaboost works with noisy data and also provides high efficacy in a complicated environment like the Internet of Things (IoT). Moreover, the main advantage of Adaboost is its ability to capture the minute features that define the difference in attack detection probability when compared with normal traffic detection [63]. Adaboost quickly reduces errors and generates a strong classifier, making it an appropriate solution regarding the hardware restrictions in an IoT network environment

### 3.10. Justification of Utilizing the Models

The rationale for employing “Logistic Regression” is its simplicity, interpretability, and efficiency as a solution for binary classification tasks characterized by a distinct linear boundary separating attack from normal activity [61]. The rationale for employing “Decision Trees” is their capability to manage both qualitative and numerical data, which is advantageous for identifying distinct patterns of malevolent activity [64]. The “Random Forest”, as an ensemble method, mitigates overfitting and enhances generalization, hence facilitating the identification of nuanced and diverse patterns in attack behavior [65]. Moreover, Neural Networks were utilized due to their ability to identify intricate patterns and correlations, rendering them ideal for detecting advanced, loop-based attacks with nonlinear attributes [66]. The rationale for adopting SVM is its efficacy in delineating classes with a distinct margin, which is advantageous for binary categorization of normal versus malevolent activity [67]. Moreover, the rationale for employing the “K-Nearest Neighbors” algorithm is its ability to identify local anomalies by examining the “neighbors” of IoT data points, making it suitable for real-time anomaly identification. Another rationale for employing “Naive Bayes” is its speed and efficiency, making it appropriate for applications where simplicity and rapid decision-making are essential, such as real-time detection in resource-constrained IoT devices [68]. The rationale for employing “XGBoost” is in its integration of boosting and regularization, rendering it highly effective for managing extensive datasets with numerous features, as typically encountered in IoT data. Ultimately, each of these algorithms possesses strengths tailored for identifying malicious patterns, utilizing various factors such as complexity, computing efficiency, or capacity to manage large-scale data characteristic of IoT contexts [69].

### 3.11. Computational and Energy Cost Associated to the Models

Typically, other operating metrics related to the models, such as computational and energy cost are crucial. The simple models like “Logistic Regression” and “Naive Bayes” are computationally and energy-efficient because they rely on straightforward mathematical operations. However, moderate complexity models like “Decision Trees” and “K-Nearest Neighbors” require more resources, especially on large datasets. High complexity models such as “Neural Networks”, “SVMs (with non-linear kernels)”, and ensemble methods like “Random Forest” and “XGBoost” are computationally intensive, making them energy-intensive as well.

The training of these models generally consumes more energy than inference. Models like KNN, however, reverse linear pattern, as training is merely data storage, but inference requires calculating distances to all stored data points. Inference cost is relatively low for linear models (Logistic Regression, Naive Bayes), where each prediction involves simple calculations. In contrast, KNN and some deep learning models can be costly at inference due to the need for extensive computation.

Another important aspect of this lies with “Large Datasets”. Algorithms like XGBoost and Random Forest are well-suited for large datasets but become computationally demanding. Conversely, Naive Bayes remains efficient with large datasets. High-dimensional data can make even simple models like Logistic Regression and SVM computationally expensive due to the increase in mathematical operations required for each feature.

Some models (e.g., Neural Networks, XGBoost) benefit from specialized hardware like GPUs or TPUs, which can speed up training and reduce overall runtime. However, these accelerators consume more energy than CPUs, potentially leading to high energy costs despite faster computation. Energy costs become important when deploying models in resource-constrained environments (e.g., edge devices, mobile applications). Lightweight models (Logistic Regression, Naive Bayes, simple Decision Trees) are generally preferred for these applications, while energy-intensive models (Neural Networks, SVMs with non-linear kernels) are better suited for high-performance computing environments where energy resources are less constrained.

## 4. Research Methodology

This study’s experimental technique follows the conventional framework for machine learning experimental analysis. First, the research analyzes the models and do any necessary pre-processing. Afterwards, the model was assessed. In this part, we detail the measures that were used to evaluate the model.

### 4.1. The Architectural Layer of the IoT and Loops in the System

The IoT has many architecture, however, the common ones are three and five-layer architecture. The three-layer architecture is the most basic type of architecture. “The perception layer”, “the network layer”, and “the application layer” are the three layers of the architecture. While the three-layer design’s “perception layer” and “application layer” are still present in the five-layer architecture, three additional architectural components—the “business layer”, “processing layer”, and “transport layer”—are added. Loop refers to the concept of iterations. Precisely, a loop is a repeating statement within a group of associated statements. The repeated behavior is usually a block of code encapsulating contents that may change in value. When the associated states change to specific conditions, infinite loops may occur. Loops are a common phenomenon in computer systems. Loops often lead to service resource exhaustion. Looping is the main concern for an IoT network as it perpetuates the occurrence of identical packets in the network. That is why this current research set out to model a detection of Loop-based attack in IoT.

### 4.2. System Model

**Figure 1:**
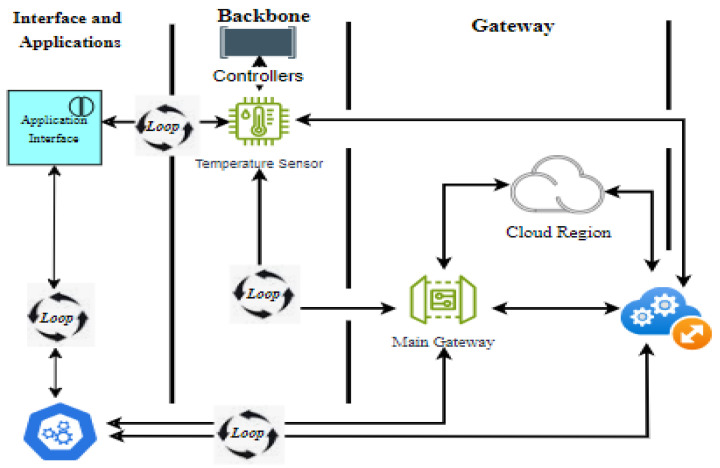
The system model associated with loop-based attack.

The system model where a loop-based attack can be executed comprise various components, including Interface and Applications, Backbone Controllers, Gateways, and Cloud Regions (see Figure 1). This architecture showcases a potential loop-based attack on an IoT system. The nature of the problem that would lead to the attacks lies with the fact that IoT devices and their connected components (such as controllers and gateways) often lack robust error-handling mechanisms, making them vulnerable to loop-based attacks. Attackers can inject malicious loops into these systems, causing components to process repetitive tasks indefinitely. Hence, the impact on “System Resources” comes from a continuous execution of loops consuming the computational and memory resources of each component. When multiple devices enter a loop simultaneously, it overwhelms the network and central components (e.g., gateways and cloud), leading to a DoS.

As seen in the model, a loop injected in one component (such as the Temperature Sensor) can propagate through controllers and the main gateway to other connected components, eventually affecting the entire network. The execution of the attacks is presented in Figure 1. The initiation of malicious Loop in a IoT system starts with the attacker targeting a vulnerable device, such as a “Temperature Sensor” in the “Backbone Controllers layer”. By exploiting weak security protocols or invalidated input, the attacker injects a command or code that initiates an infinite loop in the “Temperature Sensor”.

The propagation of the Malicious Loop within the “Temperature Sensor” begins sending continuous, repetitive data or requests due to the loop. Instead of sending valid temperature readings at intervals, it transmits excessive requests to the next component in the chain. The temperature sensor’s repetitive loop is detected by the controllers. However, due to insufficient validation or timeout mechanisms, the controllers start processing these continuous requests, entering their own loop as they become overloaded by handling the same commands repeatedly.

The controllers forward these repetitive packets to the “Main Gateway”. The gateway is responsible for managing and routing traffic, and becomes overwhelmed as it handles the excessive data load from the controllers. As the gateway processes these requests in a continuous loop, it starts consuming excessive resources, reducing its capacity to handle legitimate traffic from other devices. From the gateway, the malicious loop propagates to the Cloud Region. This cloud component, designed for large-scale data processing and analytics, becomes overburdened by the continuous looped data coming from the gateway. The cloud system, while capable of handling large data volumes, may still experience degraded performance or service disruptions due to the unexpected surge in traffic caused by the loop.

The Application Interface interacts with users and receives data from the cloud. If the interface receives repeated data due to the loop, it can display incorrect information or become unresponsive. The Backbone Controllers and Temperature Sensor serve as points of entry and propagation for the malicious loop. Vulnerabilities in these components allow the attack to initiate and propagate.

The Main Gateway acts as a central router. The gateway’s lack of validation or overload prevention mechanisms allows the malicious loop to affect other connected components. Finally, the “cloud region” receives and processes the data from the gateway. As the final destination, it becomes a bottleneck for the excessive traffic caused by the loop, impacting cloud-based analytics and storage.

### 4.3. Dataset

Datasets developed by the Czech Technical University in Prague for the purpose of studying and analyzing malware behavior, with a focus on IoT devices, were adopted for this study. Specifically, the “CTU-IoT-Malware-Capture-7-1 (“Linux, Mirai”) part of the dataset that captured for a duration of 24 h, with 11,000,000 packets, was selected for this study (see Table 1) [70].

With an emphasis on a botnet attack utilizing the Mirai malware, this particular dataset contains both benign and malicious traffic. The communication protocol used for the dataset includes TCP, UDP, and ICMP. In IoT botnet traffic, there are also protocols related to HTTP, DNS, or custom protocols used by Mirai malware.

Within the pcap file of the dataset, a loop mechanism was studied and data associated with the loop were extracted. The typical scenario associated with the extraction lasts just 10 s, and about 100,000 packets are obtained from the dataset within a certain transmission session. Although some entries are classified as benign, an analysis of the transmission sessions reveals many entries, with more than 10,000 packets per second. A total of 1,686,291 packets were transmitted during the 24-h time frame over the 4 days. Typically, the extraction of the loop within the transmission sessions lies in the use of the identifier header in the packet captured, which is a relatively unique sequential number, and duplicates should not occur. If they do, then a loop happens. However, in extensive datasets, this number may be reused, but still not for a large period of time. The research received many packets exhibiting identical IP identifying numbers, sources, and destinations. Hence, all these data were gathered.

The dataset was categorized under two classes: “Class 0: Represents normal traffic or device behavior (non-attack)”. “Class 1: Represents malicious traffic or compromised device behavior (attack)”. This is different from the original “CTU-IoT-Malware-Capture-7-1 (“Linux, Mirai”) for the fact that their original labelling was “Anomaly” or “Benign”. However, for this research, some of the malicious labels found in the dataset do not involve loops. Hence, for this current research they are categorized under benign. Hence, the anomalies or malicious behavior in IoT networks for this research refer to loops, where Class 0 and Class 1 refer to the two possible output labels or categories that a model is trying to distinguish between loops and the normal. As a result, Class 0 typically refers to the negative class or the “normal” class. This means that instances classified as Class 0 are deemed to belong to the benign or non-malicious group of loops, whereas Class 1 refers to the positive class or the “anomalous/malicious” class loops. This class typically represents instances where the model detects an anomaly or threat of loops attack.

The dataset is divided into two partitions: training data and testing data. A total of 80% of the data are allocated for training the model, while 20% are reserved for assessing the model’s performance. The splitting of the dataset guarantees that the assessment is conducted on data that the model has not previously encountered. Finally, all the features are normalized to a comparable scale to guarantee that no single feature predominates over the others.

### 4.4. Performance Metrics

If one is comparing the predictive ability of multiple models, performance metrics serve as the lens that lets us understand how the models compare. Additionally, these metrics let us understand what is working in a particular model and how to make it better. This breaks down such performance metrics in machine learning: namely accuracy, precision, recall, F1-score, area under curve (AUC), mean absolute error, mean squared error, and the coefficient of determination.

Accuracy can basically be defined as the ratio of the number of correct predictions over the total number of predictions. Accuracy is one of the most straightforward performance metrics in machine learning. It simply measures the proportion of correct predictions made by a model. Mathematically, accuracy is the ratio of the number of correct predictions to the total number of predictions as shown in Equation (8):

(8) $$\mathrm{Accuracy}=\frac{\mathrm{TP}+\mathrm{TN}}{\mathrm{TP}+\mathrm{TN}+\mathrm{FP}+\mathrm{FN}}$$

where TP is “True Positive”, TN is “True Negative”, FP “False Positive”, and FN “False Negative”.

Precision is another important metric used to measure a model’s accuracy. Precision refers to the accuracy of the positive predictions that it makes. The ratio that precision represents is calculated as true positives divided by the sum of true positives and false positives. This metric is very useful in cases where making positive predictions has very bad outcomes. The formula to calculate precision is presented in Equation (9):

(9) $$\mathrm{Precision}=\frac{\mathrm{TP}}{\mathrm{TP}+\mathrm{FP}}$$

Recall is one of the most critical indicators of how effectively a model is able to identify relevant instances. Unlike precision, which focuses on the response aspect of suggesting only relevant instances, recall concentrates on the technique aspect of not leaving any relevant instance behind. Mathematically, it can be written as the proportion between the true positives and the sum of true positives with the false negatives (see Equation (10)):

(10) $$\mathrm{Recall}=\frac{\mathrm{TP}}{\mathrm{TP}+\mathrm{FN}}$$

F1 score is a measure of how accurate a model is, as well as a measure of the reliability of the model. It mixes both recall and precision (See Equation (11)). F1 score is the harmonic mean of precision and recall.

(11) $$F1\text{-}\mathrm{score}=\frac{\mathrm{TP}}{\mathrm{TP}+\frac{1}{2}\left(FP+FN\right)}$$

## 5. Experimentation and Presentation of the Results

All models deployed in this study have been initialized, as detailed in Section 3. Developing the code required to create and configure various models aimed at predicting the occurrence of Unusual Repetitive Patterns of Behavior Indicative of a Loop-based Attack in IoT is an essential phase in the initialization of machine learning models. Both the training and the prediction were addressed. The training data, comprising the subset of “CTU-IoT-Malware-Capture-7-1” already allocated for model instruction, are employed in the training of each specific model.

XGBoost (0.9251) and Logistic Regression (0.9104) have the highest accuracy scores, which is typical for these models, as they often perform well on structured datasets. Random Forest, AdaBoost, and SVM have slightly lower accuracy, which is consistent with how they might behave on binary classification problems, particularly if there is class imbalance or noise in the data (see Table 2).

In most cases, precision for Class 1 (the minority class, assuming this represents the malicious class) is higher than for Class 0, which suggests that the models are better at identifying the attack class. However, XGBoost stands out with notably high precision values for both classes (0.9419 for Class 0 and 0.9657 for Class 1), which is expected as XGBoost often excels in handling complex classification problems with many features.

The recall values vary, with most models having higher recall for Class 1. This suggests that the models are prioritizing identifying malicious activity over benign activity, which is a common trade-off in anomaly detection scenarios. For example, the Random Forest has a recall of 0.9698 for Class 1, showing that it is highly effective at capturing attacks, though it has lower recall for Class 0 (0.7652).

The F1-score (Class 1) is notably higher for models like XGBoost (0.9624) and Random Forest (0.9542), which aligns with their strong performance in both precision and recall for detecting attacks. This suggests these models are well suited for balancing false positives and false negatives in IoT security or other anomaly detection applications.

On the other hand, Logistic Regression and Decision Trees also perform well, but their F1-scores are slightly lower than ensemble methods like XGBoost and Random Forest, as expected.

Logistic Regression: Performs well with an accuracy of 0.9104, which is strong for a simple linear model. However, the F1-scores indicate that this model may struggle slightly with Class 1 compared to more complex models.

XGBoost: as expected, XGBoost achieves the best performance across most metrics (accuracy, precision, recall, and F1-score), showing its strength in handling large, feature-rich datasets and complex decision boundaries.

SVM: SVM shows moderate performance in terms of accuracy (0.8857) and precision for Class 1 (0.8969). This is consistent with SVM’s tendency to perform well on linear separable data but sometimes struggle with complex, non-linear patterns.

K-Nearest Neighbors (KNN): with a lower accuracy (0.8107) and F1-scores, KNN tends to be less efficient in larger datasets or when there is significant noise, which might explain its relatively lower performance.

Naive Bayes: This algorithm performs reasonably well given its simplicity, though its accuracy and precision are lower than more advanced models, reflecting its limitation in handling feature dependencies.

These results are largely consistent with how the models typically perform, especially in binary classification tasks like anomaly detection in IoT. XGBoost and Random Forest are often superior in such scenarios due to their ability to handle complex data patterns and avoid overfitting. However, simpler models like Logistic Regression and Decision Trees still perform reasonably well.

**Figure 2:**
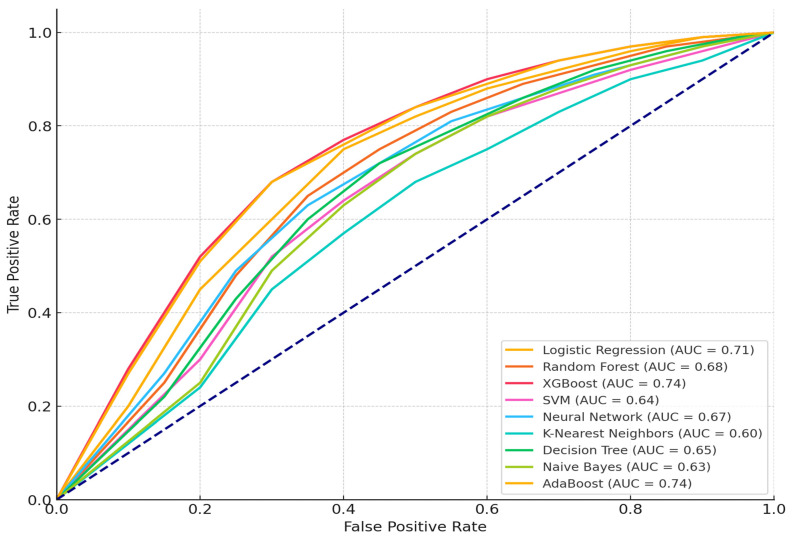
The AUC distribution of the performance of the model before optimization.

Figure 2 displays the Receiver Operating Characteristic (ROC) curve for each model based on the supplied data. Each curve illustrates the trade-off between the true positive rate (sensitivity) calculated mathematically by Equation (12):

(12) $$True\;Postive\;Rate=\frac{\mathrm{TP}}{\mathrm{TP}+FN}$$

where TP is “True Positive” and TN is “True Negative”. The false positive rate for each model, offering insight into their categorization efficacy, is also calculate by Equation (13):

(13) $$False\;Postive\;Rate=\frac{\mathrm{FP}}{\mathrm{FN}+TN}$$

The AUC is specified in the legend for each model, indicating their overall efficacy in differentiating across classes. It is measured by Equation (14):

(14) $$AUC=\frac{Number\;of\;Concordant\;Pairs}{\mathrm{Total}\;\mathrm{Number}\;\mathrm{of}\;\mathrm{Positive}-\mathrm{Negative}\;\mathrm{Pairs}\;}$$

AUC = 1.0: complete class distinction. AUC < 1.0 (0.5): shows a high capacity for discrimination. The blue dotted line in a ROC curve represents the “line of no discrimination”. This line, which runs at a 45-degree angle from the bottom left to the top right (diagonal line), corresponds to a model that makes random predictions, with an AUC of 0.5. It serves as a baseline for comparison. Models with curves above this line have predictive power, while curves at or below it indicates poor or random performance”

XGBoost AUC ≈ 0.92 demonstrates excellent discriminative ability, effectively distinguishing between normal and malicious activities, whereas the Decision Tree’s AUC ≈ 0.75 demonstrates the lowest performance among the models, suggesting overfitting or limited ability to capture complex patterns.

Based on the ROC-AUC Scores, XGBoost stands out as the best-performing model, demonstrating the highest ability to distinguish between normal and malicious activities in IoT contexts. Its integration of boosting and regularization techniques allows it to handle extensive datasets with numerous features effectively. However, AdaBoost and Random Forest also perform exceptionally well, making them reliable choices for real-time anomaly detection in IoT environments.

The research further considers Fine-Tuning hyperparameter optimization to enhance model performance. Cross-Validation was implemented to ensure model generalization. The results from the hyperparameter tuning using GridSearchCV show a noticeable improvement in model performance across various metrics, especially Cross-Validation Accuracy, Test Accuracy, and other performance metrics such as Precision, Recall, and ROC-AUC score (see Table 3). GridSearchCV is a plugin obtained from Python library scikit-learn, through “import GridSearchCV” and is used for hyperparameter tuning.

Logistic Regression has shown a Cross-Validation Accuracy as high as 0.9507, but the Test Accuracy dropped to 0.8153, indicating potential overfitting. Precision for Class 1 is solid (0.8135), and Recall for Class 1 is particularly high (0.951), suggesting that the model is effective at identifying true positives (attacks), although some true negatives might be misclassified. The ROC-AUC Score is strong at 0.9131, demonstrating good overall discriminative ability. Both Cross-Validation Accuracy (0.9816) and Test Accuracy (0.8701) are high, reflecting that the model generalizes well. Precision and Recall for Class 1 are excellent (0.8751 and 0.963, respectively), showing its capability to handle attack detection effectively. The ROC-AUC Score is 0.9037, indicating that this model performs reliably across thresholds.

XGBoost achieves a high Cross-Validation Accuracy of 0.9885 and Test Accuracy of 0.8701, indicating consistency in performance. Precision (Class 1: 0.8722) and Recall (Class 1: 0.944) are strong, though slightly lower than Random Forest in precision. The ROC-AUC Score (0.9207) is among the highest, emphasizing its ability to manage imbalanced datasets with many features effectively.

SVM shows solid performance with Cross-Validation Accuracy (0.9541) and Test Accuracy (0.8427). Precision (0.8649 for Class 1) and Recall (0.943 for Class 1) reflect that it handles classification well, though it may misclassify more negative instances (Class 0). The ROC-AUC Score (0.9122) indicates overall strong performance.

Neural Network shows a Cross-Validation Accuracy (0.9542) and Test Accuracy (0.8564) show that the neural network is robust but might require additional tuning for more generalization. Precision for Class 1 (0.8945) and Recall (0.903) indicate it is good at detecting attacks, though Precision for Class 0 is relatively lower. The ROC-AUC Score is 0.9037, which is competitive, showing that it performs well on overall classification tasks.

KNN underperforms compared to other models, with the lowest Test Accuracy (0.7742) and ROC-AUC Score (0.8161). Precision and Recall for Class 0 and Class 1 suggest that KNN is less reliable for detecting attacks and suffers from sensitivity to large datasets and noise.

Decision Tree shows a Cross-Validation Accuracy is relatively high at 0.9747, but Test Accuracy (0.8279) suggests some overfitting. Recall for Class 1 (0.923) is solid, though Precision for Class 0 (0.7991) suggests room for improvement in detecting non-attacks. ROC-AUC Score is 0.7477, indicating that Decision Tree is less effective than ensemble methods.

Naive Bayes shows a Cross-Validation Accuracy (0.8856) is reasonable, but the Test Accuracy (0.8153) shows that Naive Bayes struggles compared to other models. Precision (0.8748 for Class 1) and Recall (0.843 for Class 1) are acceptable, but overall performance is lower due to the model’s simplicity. A ROC-AUC Score of 0.8748 reflects a balanced but less effective performance compared to complex models like Random Forest and XGBoost.

AdaBoost shows Cross-Validation Accuracy is extremely high (0.9987), and a Test Accuracy of 0.8427 suggests AdaBoost generalizes well, although not as well as Random Forest or XGBoost. A Precision of 0.8743 and Recall of 0.943 for Class 1 show that AdaBoost performs strongly on attack detection. A ROC-AUC Score of 0.9165 highlights its reliability in distinguishing between classes.

XGBoost and Random Forest are the standout models, offering the highest performance metrics across precision, recall, and ROC-AUC scores, making them the most reliable for anomaly detection in IoT or security applications. Logistic Regression and SVM provide solid performance but may require additional fine-tuning to handle non-linear and complex patterns found in security data. Naive Bayes and KNN are outperformed by more complex models, showing their limitations in handling more intricate or noisy datasets.

**Figure 3:**
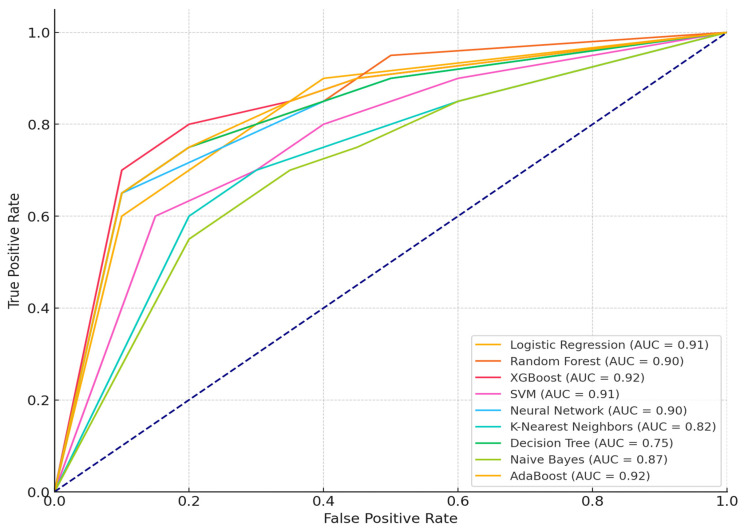
AUC performance after fine tuning the model.

The notable improvements XGBoost and AdaBoost have the highest AUC (0.92), indicating they have the best trade-off between true positive and false positive rates. XGBoost consistently shows high AUC values in both the current and previous results (see Figure 3). However, the precision and recall for Class 1 have slightly improved, indicating that tuning parameters have resulted in better performance, especially in handling false positives and false negatives. Logistic Regression, SVM, and Random Forest also perform well with AUC scores around 0.91 and 0.90. K-Nearest Neighbors and Decision Tree have relatively lower AUC scores, indicating they may not perform as well in distinguishing between classes compared to the other models. These curves visually represent how well each model balances sensitivity (True Positive Rate) and specificity (False Positive Rate), with higher curves (closer to the top left) indicating better model performance

## 6. Discussion and Future Directions

**Figure 4:**
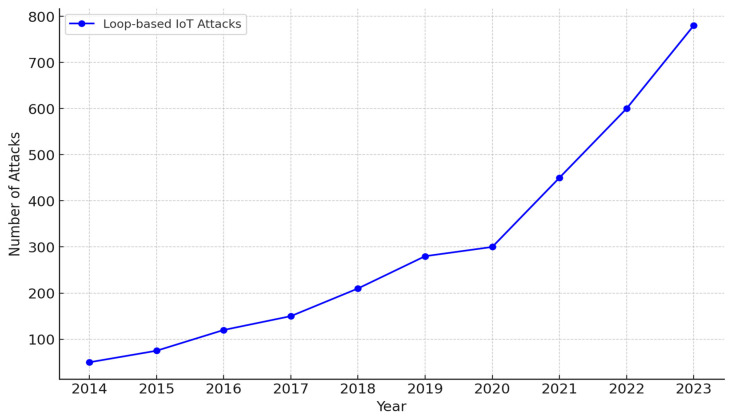
The frequencies of loop-based IoT attacks.

In this paper, it was established that in an IoT attack dataset, a loop-based attack can be extracted in order to explore the suitable machine learning model that is deemed fit to detect them. The central area of this research focuses on the synthesizing of dataset methods to effectively and efficiently detect loop-based malicious attacks in IoT networks. This is necessary based on the fact that loop-based IoT attacks over the last 10 years (2014–2024) were indicated to have a significant increase (see Figure 4) [71].

The academic community is aggressively tackling the growing threat of loop-based attacks and other vulnerabilities in the Internet of Things (IoT) through a variety of tactics, with an emphasis on both theoretical developments and practical implementations. That is why this current research first analyzes the existence and working mechanisms of loops in IoT network clusters, which leads to resource-starved nodes in the network.

The results of this study highlight that, within a known dataset that is intended to measure malicious intent, loop-based attack signatures can be derived. The importance of this lies in the fact that Loop-based attacks are very effective in causing mismanagement problems in the IoT environment as they shape the behavior of the infected IoT device by repeating actions. The repetition of actions can be used to make the IoT device generate fake sensor or feedback data to change its behavior or to reconnect with the Command and Control server or peer IoT devices for possible updates. Loop-based attacks can be implemented by using various repetitive actions, which change the behavior of the IoT device and force it to perform activities causing insecurity in the IoT systems by embedding it into the normal behavior of the IoT devices. A deep-dive analysis of repetitive actions can create an understanding of these attacks’ potential contribution to causing inconvenience in the IoT space and finding out their similarities or differences, if any. This repetitive action consists of malicious commands that are redirected to the IoT device using stolen or implanted identity that is not supposed to be directed to by the user of the IoT device.

Another important implication of this research lies with one of the features of loop-based malicious attacks. That is, sending a fake message to the victim system continuously. This fake message is then able to manipulate the system to make entirely incorrect decisions. Critical to this situation lies with an intruder in a home automation system who sends fake data so that power-hungry devices are started. In a city-scale IoT system, this can potentially damage part of the existing critical infrastructure. In computers, the usual technique used to achieve a computer operating in an infinite-state event loop is by redirecting its execution point to the beginning of an instruction or to an unexpected memory address to start execution of an unrelated set of machine instructions. In IoT systems, attackers with deep technical knowledge of the network layer, communication protocol characteristics, application layer credentials, and sensor message structure are able to create a fake IoT message that is indistinguishable from a true message. The results of this research offer a solution to prevent these scenarios.

This research’s impacts on the theory revealed that Loop-based attacks that can cause a range of disruptions to the targeted or interconnected IoT systems, both in terms of reliability and performance, which can be detected. Such attacks, if undetected, can cause an immediate and complete or partial failure of the system. This usually has an impact on the quality of service provided by the system or the cloud-based components. The impacts have also been evaluated for datasets especially focused on a deterministic dependence between the right side of the computational matrix and some specific IoT building indicators.

This research finally compares the proposed method with some existing detection frameworks and presented the comparative findings in Table 4. The best-performing paper from the previous studies in terms of Accuracy, Precision, Recall, and F1 Score is the work by Brindha Devi et al. [30] with an Accuracy: 95%, Precision: 94%, Recall: 93%, and F1 Score: 93.5%. The optimized learning model in this study achieves superior performance metrics, indicating its effectiveness in detecting and mitigating IoT attacks. The high precision and recall suggest that the model can reliably distinguish between normal and malicious traffic, minimizing false positives and negatives. This makes the system particularly valuable for real-time applications in IoT environments where accurate and timely threat detection is critical to maintaining system functionality and security. When comparing the best-performing study in terms of accuracy, precision, recall, and F1-score, this current research outperforms the previous research studies with the “XGBoost” model.

## 7. Conclusions

Many consumer, industrial, and military applications use the technology known as the Internet of Things (IoT) extensively. The Internet of Things, on the other hand, presents possible security weaknesses. It is unfortunate that the conventional defense system is not capable of dealing with this specific danger. Given the significant risk of breach that Internet of Things devices face, it would be prudent to consider the attack detection component. On the other hand, the intrinsic limits of the Internet of Things (IoT), which include battery life, sensor capabilities, and processing power, frequently render the commonly used detection methods inefficient. Examples of these methods include signature-based detection and anomaly-based detection. In this work, we discovered loop cases from the transmission session dataset of “CTU-IoT-Malware-Capture-7-1”, labeled as “Linux, Mirai”, and constructed a loop-based detection machine learning algorithm. This research project utilized a total of nine different machine learning models to demonstrate detection through the examination of loop patterns in datasets. According to the findings of this study, the XGBoost model is capable of achieving exceptional detection performance, as evidenced by its “Accuracy: 98.85%”, “Precision: 96.57% (Class 1)”, “Recall: 96.72% (Class 1)”, and “F1-Score: 96.24%”. Because the XGBoost model performed exceptionally well across all criteria, it is clear that it is capable of managing enormous datasets that are associated with the Internet of Things. Its high degree of accuracy and substantial generalization capability enable it to detect sophisticated and diversified patterns of harmful activity in Internet of Things networks. In addition, its precision and recall metrics provide further evidence of its effectiveness in distinguishing between attack and normal operations. As a result, it reduces the possibility of false positives and negatives, making it an excellent option for real-time Internet of Things threat detection.

## Figures and Tables

**Table 1:** Dataset labels distribution.

Label	Flows
Benign	75,955
C&C-HeartBeat	5778
DDoS	39,584
Okiru	11,333,397

**Table 2:** Performance values for all the model.

Model	Accuracy	Precision (Class 0)	Precision (Class 1)	Recall (Class 0)	Recall (Class 1)	F1-Score (Class 0)	F1-Score (Class 1)
Logistic Regression	0.9104	0.8978	0.9125	0.8617	0.9485	0.7234	0.8918
Random Forest	0.8911	0.9108	0.9014	0.7652	0.9698	0.7439	0.9542
XGBoost	0.9251	0.9419	0.9657	0.8737	0.9672	0.7657	0.9624
SVM	0.8857	0.8194	0.8969	0.8719	0.8715	0.7412	0.8614
Neural Network	0.8757	0.8527	0.8987	0.8753	0.8982	0.7455	0.8895
K-Nearest Neighbors	0.8107	0.8414	0.8149	0.7984	0.9178	0.7085	0.8925
Decision Tree	0.8714	0.9014	0.8995	0.8679	0.9542	0.7074	0.8919
Naive Bayes	0.8347	0.7348	0.8978	0.8975	0.8849	0.7272	0.8985
AdaBoost	0.8821	0.8736	0.8784	0.7892	0.9541	0.7421	0.8938

**Table 3:** Performance after fine tuning the model.

Model	Cross-Validation Accuracy	Test Accuracy	Precision (Class 0)	Precision (Class 1)	Recall (Class 0)	Recall (Class 1)	ROC-AUC Score
Logistic Regression	0.9507	0.8153	0.7571	0.8135	0.7262	0.9521	0.9131
Random Forest	0.9816	0.8701	0.8973	0.8751	0.7617	0.9603	0.9037
XGBoost	0.9885	0.8701	0.8961	0.8722	0.7771	0.9414	0.9207
SVM	0.9541	0.8427	0.8374	0.8649	0.7421	0.9423	0.9122
Neural Network	0.9542	0.8564	0.7871	0.8945	0.8153	0.9043	0.9037
K-Nearest Neighbors	0.9337	0.7742	0.7155	0.8423	0.7123	0.9033	0.8161
Decision Tree	0.9747	0.8279	0.7991	0.8531	0.7912	0.9213	0.7477
Naive Bayes	0.8856	0.8153	0.6971	0.8748	0.7819	0.8413	0.8748
AdaBoost	0.9987	0.8427	0.8349	0.8743	0.7759	0.9433	0.9165

**Table 4:** The Comparative performance measurement.

Source	Dataset	Model	Accuracy	Precision	Recall	F1 Score
[30]	IoT traffic and attack datasets	Optimized Deep Learning	0.9521	0.9432	0.9303	0.9351
[31]	IoT sensor datasets	Unsupervised Learning	0.9132	0.9001	0.8901	0.8952
[32]	IoT routing datasets	DAIR-MLT	0.8912	0.8821	0.8703	0.8751
[33]	Edge computing IoT datasets	Reinforcement Learning	0.9301	0.9221	0.9123	0.9150
This study	Custom IoT Dataset	XGBoost	0.9885	0.9657	0.9672	0.9624

## Data Availability

Data will be shared on demand.
